# 

**Published:** 1822-12-01

**Authors:** 


### Contents

OF

No. 11. Vol. III. for DECEMBER, 1822.

ART. I.

Dr. LALLEMAND's Researches on the Pathological Anatomy of the Brain and its Coverings - - - - - - - - - 465

II.

NEURALGIA.

1. Dr. HUTCHINSON on Neuralgia Spasmodica, (2d edition)
2. Dr. YEATS' History of a Case of Neuralgia in the Lower Extremity - - - - - - - - - - - - - - - -
3. Dr. SHIRLEY PALMER's Observations on Neuralgia - - } 495

III.

CHLORINE AND NITRO-MURIATIC ACID.

1. Mr. WALLACE on the Medical Powers of Chlorine - -
2. Mr. COYNE on the Nitro-Muriatic Acid Bath - - - - } 510

IV.

A Collection of Surgical Memoirs. By Baron D. J. LARREY 523

V.

Mr. JEFFREYS' Cases in Surgery, from the Records of the St. George's and St. James's Dispensary - - - - - 535

VI.

Mr. COOKE's Abridgment of Morgagni on the Seats and Causes of Diseases - - - - - - - - - - - - - - 546

VII.

The Study of Medicine. By JOHN MASON GOOD, M. D. Art. 1. On the Class CŒLIACA, or Diseases of the DIGESTIVE ORGANS - - - - - - - - - - - - - - 574

VIII.

A Review of the General Principles of Physiology. By Dr. W. PHILIP - - - - - - - - - - - - - - - - - 596

IX.

Sir ASTLEY COOPER on Dislocations and Fractures of the Joints. (First Analytical Article.) - - - - - - - 612

### X.

### Quarterly Periscope;

OR,

### SPIRIT OF THE PUBLIC JOURNALS.

| ART. | | Page |
|---|---|---|
| 1. | Series of Metastatic Inflammations | 641 |
| 2. | Dr. Gregory on Cynanche Cellularis | 644 |
| 3. | Dr. Forbes on Variola and Vaccina | 646 |
| 4. | Mr. Moir on Puerperal Fever | 648 |
| | Editor's Case of Ditto | 650 |
| 5. | Dr. Forbes on Tar Vapour | 651 |
| 6. | Dr. Dewees on simulated Pregnancy | 652 |
| 7. | Drs. Howison and Johnson on Renal Dropsy | 653 |
| 8. | Singular Case of Chorea | 659 |
| 9. | Kergaradec on the Stethoscope | 661 |
| 10. | Dr. Gooch on Uterine Hæmorrhage | 662 |
| 11. | Dr. Smith on Iritis | 666 |
| 12. | Hypochondriasis and Suicide | 667 |
| 13. | Mr. Dunn on Compound Fractures | 669 |
| 14. | Mr. Fosbroke's Case of Scrophulous Disease | 671 |
| 15. | Curious tendency to Suicide | 672 |
| 16. | Dr. Robertson on Retroversion of the Uterus | 673 |
| 17. | Dr. Cooper's Test for Arsenic | 674 |
| 18. | Curious Case of Hepato-pulmonic Disease | 675 |
| 19. | Dr. Shearman on Epilepsy | 677 |
| 20. | Stricture of the Œsophagus | 677 |
| 21. | Mr. Green's Extraction of a Fœtus | 677 |
| 22. | On Puerperal Fever | 678 |
| 23. | Purpura Hæmorrhagica | 679 |
| 24. | On large Doses of Tartrite of Antimony | 680 |
| 25. | Mr. Liston's Case of Cystitis | 681 |
| 26. | Elongated Uvula | 681 |
| 27. | Experiments on the Spinal Nerves | 682 |
| 28. | Extraction of Poisons from the Stomach | 683 |
| 29. | Curious Attempts at Suicide | 684 |
| 30. | Mr. Bampfield on Spinal Distortion | 686 |
| 31. | Dr. Sutton on Limitation of Emetics | 686 |
| 32. | Mr. Churchill on Gonorrhœa | 686 |

### XI.

Bibliographical Record - - - - - - - - - - - - - 687

### XII.

EXTRA LIMITES.

| | | Page |
|---|---|---|
| Art. I. | Dr. Wilson on Morbid Sympathies | 691 |
| II. | Dr. Johnson's Case of Mr. Knox | 694 |
| III. | Mr. Swan on Hare-Lip | 696 |

### XIII.

Correspondence, Intelligence, &c. - - - - - - - - - 697

